# Dietary Chitosan Supplementation Increases Microbial Diversity and Attenuates the Severity of *Citrobacter rodentium* Infection in Mice

**DOI:** 10.1155/2016/9236196

**Published:** 2016-09-28

**Authors:** Guiping Guan, Hongbing Wang, Shuai Chen, Gang Liu, Xia Xiong, Bie Tan, Veeramuthu Duraipandiyan, Naif Abdullah Al-Dhabi, Jun Fang

**Affiliations:** ^1^College of Bioscience and Biotechnology, Hunan Agricultural University, Changsha, Hunan 410128, China; ^2^Key Laboratory of Agroecological Processes in Subtropical Region, Institute of Subtropical Agriculture, Chinese Academy of Sciences, Hunan Provincial Engineering Research Center of Healthy Livestock, Scientific Observing and Experimental Station of Animal Nutrition and Feed Science in South-Central, Ministry of Agriculture, Hunan Co-Innovation Center of Animal Production Safety, Hunan 410125, China; ^3^Hunan Institute of Animal and Veterinary Science, Changsha, Changsha 410131, China; ^4^Department of Botany and Microbiology, Addiriyah Chair for Environmental Studies, College of Science, King Saud University, P.O. Box. 2455, Riyadh 11451, Saudi Arabia

## Abstract

C57BL/6 mice were tested in order to investigate the effects of dietary chitosan (COS) supplements on intestinal microflora and resistance to* Citrobacter rodentium* infection. The findings reveal that, after consuming a 300 mg/kg COS diet for 14 days, microflora became more diverse as a result of the supplement. Mice receiving COS exhibited an increase in the percentage of Bacteroidetes phylum and a decrease in the percentage of Firmicutes phylum. After* Citrobacter rodentium* infection, the histopathology scores indicated that COS feeding resulted in less severe colitis. IL-6 and TNF-*α* were significantly lower in colon from COS-feeding mice than those in the control group. Furthermore, mice in COS group were also found to experience inhibited activation of nuclear factor-kappa B (NF-*κ*B) in the colonic tissue. Overall, the findings revealed that adding 300 mg/kg COS to the diet changed the composition of the intestinal microflora of mice, resulting in suppressed NF-*κ*B activation and less production of TNF-*α* and IL-6; and these changes led to better control of inflammation and resolution of infection with* C. rodentium*.

## 1. Introduction

*Citrobacter rodentium*, a mucosal pathogen found in mice, and enteropathogenic* Escherichia coli* (EPEC) and enterohaemorrhagic* E. coli* (EHEC), enteric pathogens found in humans, create attaching and effacing (A/E) lesions that result in the colonization of mucosa within the intestinal tract [1, 2]. The intestinal epithelium becomes infiltrated by bacterial attachments, the brush border microvilli become depleted, and pedestal-shaped structures begin to form beneath the adherent bacterium [3]. Colonic tissue changes comparable to EHEC and EPEC infection, along with a rise in the production of inflammatory cytokine and leukocyte infiltration, are prompted by A/E pathogens following the colonization of the colonic epithelium [4].* C. rodentium* amongst mice populations has been used by many researchers to represent intestinal* E. coli* since intestinal EPEC or EHEC does not infect mice.

A number of significant intestinal disorders found in humans are also frequently modelled by* C. rodentium* in mice, such as colon tumorigenesis, ulcerative colitis, and Crohn's disease [5]. Colitis develops in mice that have been infected with* C. rodentium*, which results in the bacteria becoming overabundant and the mice's natural microbiota reducing in variety and quantity [6]. When infected, 1–3% of the mice's intestinal microbiota becomes* C. rodentium* [7], whilst the colon comes under attack by 10^9^ colony forming units (CFUs) per g [8]. The genetic makeup of the mouse impacts the formation of* C. rodentium*-induced colitis, with mice such as C3H/HeJ and FVB/N being prone to develop colitis as a result of the infection and mice such as CD-1 and C57BL/6 being considered amongst those that are not prone to develop colitis and are only prone to subclinical symptoms [9]. Vulnerability to contracting* C. rodentium*, as well as tendency to show a certain immune response, has been found to be highly related to intestinal microbiota composition [10].

The human intestinal tract houses 500–1,000 species of microbiota at a total quantity of almost 100 trillion [11]. Various factors, including location and diet, have a significant impact on the metagenome, although these factors do not have the same effect on a single organism's genome. Pathogen displacement, the extraction of energy such as SCFAs from nondigestible dietary substrates, and the development of the immune system all depend on intestinal microbiota. Significant changes in the natural microbiota homeostasis have been linked to various illnesses in humans [12].

COS is among the most plentiful polysaccharides found in insects, fungi, squid, oysters, krill, clams, and shellfish. It is a natural N-deacetylated derivative of chitin [13]. Many researchers have explored the ways in which the expression of Th1 and Th2 cytokines is impacted by COS [14]. In pigs [14], mice [15], rats [16], and fishes [17], COS serves as a regulator of the immune response. However, little is known about how intestinal microbiota is impacted by dietary COS despite a small number of studies attempting to study this in pigs and chickens [18]. It would be useful to gain insight into whether it is via the intestinal microbiota that COS is able to perform its advantageous biological functions, since it is already known that a number of biological functions are impacted by the intestinal microbiota [19, 20]. Nonetheless, at present, few findings exist on the topic of* C. rodentium*, intestinal microbiota, and the effects of COS supplements on them. Thus, the objective of this study is to evaluate the effects of COS supplementation on microbial flora composition changes, proinflammatory molecule reduction (and/or anti-inflammatory molecule increase), and* C. rodentium* infection.

## 2. Materials and Methods

### 2.1. Mice and Diet

This study used male C57BL/6 mice aged 6–8 weeks old, bred and reared at Hunan Agricultural University by breeders from China's Changsha-based SLAC Laboratory Animal Center. The reason for using only male mice was that sex and maternal factors have been shown to impact microflora composition. Hunan Agricultural University's Animal Care and Use Committees provided approval for the experiments. The mice were provided plentiful water and a normal diet [21], and they were kept individually in animal colonies that were free from pathogens. The colonies were kept at a temperature of 25°C, relative humidity of 53%, and an equal balance of 12 hours of dark/light. The mice were assigned at random to the COS and control groups after being housed in the colonies for three days, with 20 mice in each group. A normal rodent diet [21] was given to the control group. 300 mg/kg COS was added to the standard diet given to the COS group, which was consumed for 14 consecutive days. COS has a 6-sugar unit of N-acetyl glucosamine with *β*-(1–4)-linkages [8], an average molecular weight of less than 1,000 Da, and a degree of deacetylation over 95% and is free of endotoxins. The COS provided to the mice in this study was provided by the Chinese Academy of Sciences' Dalian Chemical and Physical Institute. The findings of an earlier research paper [14] were used as a guideline for the duration and amount of COS given. Throughout the experiment, the mice's weight gain, water consumption, and food consumption were recorded. Until being ready for analysis, samples were kept at −80°C.

### 2.2. *C. rodentium* Infection and Monitoring

After giving either the basal or COS diet for a period of 14 days, 10 mice in each group were infected with* C. rodentium*. CO_2_ asphyxiation was administered to all mice on D7 after infection. Samples of the mice's feces, tissue, and colonic content were gathered. Bacteria were grown in Luria Bertani broth (0.05 g/L nalidixic acid/mL) overnight. Centrifugation was used on the cultures and the pellet produced was resuspended in sterile phosphate-buffered saline (PBS). This resulted in a concentration of 5 × 10^9^ CFU/mL. Mice were then challenged at 9 am and 5 × 10^9^ CFU* C. rodentium* were orally administered to the mice using the gavage process. In order to prevent shedding mice from spreading infection, all mice were kept separately when infected with* C. rodentium*. The mice were fed with the same basal or COS diet for 7 days, respectively. Feces collection was conducted on D7 after infection, at which point they were also weighed and suspended in PBS. Serial dilutions were plated onto plates containing nalidixic acid. After a period of 24 hours, bacterial colonies were counted. Each dilution was grown at 37°C overnight using the pour plate method in MacConkey agar supplemented with the antibiotic kanamycin (40 *μ*g/mL) in order to compute only* C. rodentium*.

### 2.3. Colon Tissue: Histological Analysis

The colon of each mouse was removed and fixed in 10% formalin. Hematoxylin and eosin were used to stain and prepare paraffin-embedded sections. Six criteria (edema, ulcers, erosion, inflammation, goblet cell hyperplasia, and cryptitis) were outlined in order to histologically grade the colitis. A scale of 0–4 was used to score the lesions as follows: no epithelial thickening/colitis (0); minor epithelial cell hyperplasia/greater quantity of mucosa leukocytes (1); inflammation at numerous loci, submucosa, and mucosa infiltrated by leukocytes and/or significant epithelial cell hyperplasia (with 2-3 times' rise in crypts) (2); greater epithelial cell hyperplasia (3–10 times higher crypt count), reduction of goblet cells secreting mucin, ulceration, and/or significant leukocytic infiltration of the submucosa and mucosa (3); extremely high epithelial cell hyperplasia (10 or more times higher crypt count), crypt abscesses, and/or serious infiltration of transmural leukocytes (4).

### 2.4. 16S rDNA and Illumina MiSeq Sequencing

After giving either the basal or COS diet for a period of 14 days, feces were collected and 10 mice in each group were killed to collect colon contents. Then, feces and colon contents were used for 16S rDNA sequencing. As per the guidelines for DNA isolation, the QiagenQIAamp DNA Stool Mini Kit was used to extract DNA from luminal colon and feces contents. In order to create a baseline sample for each sample type, equal quantities of DNA were gathered from six individual mice. Primers 515F 5′-GTGCCAGCMGCCGCGG-3′ and 907R 5′-CCGTCAATTCMTTTRAGTTT-3′ (the barcode being an eight-base sequence individual to each specific sample) were used to amplify the V4-V5 region of the bacteria 16S ribosomal RNA gene by PCR. Biotree, a commercial firm based in Shanghai, carried out the general data analyses and Illumina MiSeq sequencing. Previous findings were used to guide the MiSeq PE Libraries, MiSeq sequencing, and additional analysis [22]. The corresponding authors can be contacted to request further information on references, raw data, and the sequencing run.

### 2.5. Analysis of Colonic mRNA

*IL-6* and* TNF-α* expression were incorporated in the analyses, which occurred after the collection and weighing of the proximal colon. TRIZOL regent (Invitrogen, USA) was used for the extraction of mRNA. Reverse transcription of the cDNA was conducted, and an ABI 7500 Fast RT-PCR machine (Applied Biosystems), Superscript II reverse transcriptase (Invitrogen), and oligo (dT) 20 were used to carry out real-time PCR.

### 2.6. Cytokine Measurement

A 50 mM Tris-HCl with 10 *μ*g/mL protease inhibitor solution (Sigma-Aldrich Co., USA) was used to homogenise the colonic samples over ice. Centrifugation of 20 minutes was used on the homogenates at 30,000 ×g (4°C). Following centrifugation, a Sandwich ELISA Kit (ELISA Ready-SET-GO, eBioscience, CA, USA) was used to test the supernatants for TNF-*α* and IL-6. Normalization of cytokine was carried out to match the colonic samples' protein levels.

### 2.7. NF-*κ*B (p65) Immunoblotting

The NF-*κ*B (p65) transcription factor assay kit (Cayman Chemical Company, MI, USA) was used to measure NF-*κ*B (p65) binding activity in the nuclear extracts. The proteins extracted from nuclear or cytoplasmic fractions were taken in equal quantities and then (1) divided using SDS-PAGE, (2) relocated to PVDF membranes (Millipore, MA, USA), and obstructed using 5% nonfat milk in a Tris-Tween buffered saline buffer (20 mM Tris, pH 7.5, 150 mM NaCl, 0.1% Tween-20) over a 3-hour period. Prior to conducting the analysis with Alpha Imager 2200 (Alpha Innotech Corporation, CA, USA), overnight incubation at 4°C was carried out with the primary antibodies, whilst 60-minute incubation at room temperature was carried out with the HRP-conjugated secondary antibodies. Finally, electronic quantification and normalization of signal intensity to Lamin B protein abundance were performed.

### 2.8. Statistical Data Analysis

SPSS 22.0 (Chicago, IL, USA) was used to carry out all statistical analysis. The data illustrated in this section are the means ± the standard error of the mean (SEM). Student's *t*-test was used to analyze the data between two groups. In this study, statistical significance is shown in values of *P* < 0.05.

## 3. Results

**Figure 1:**
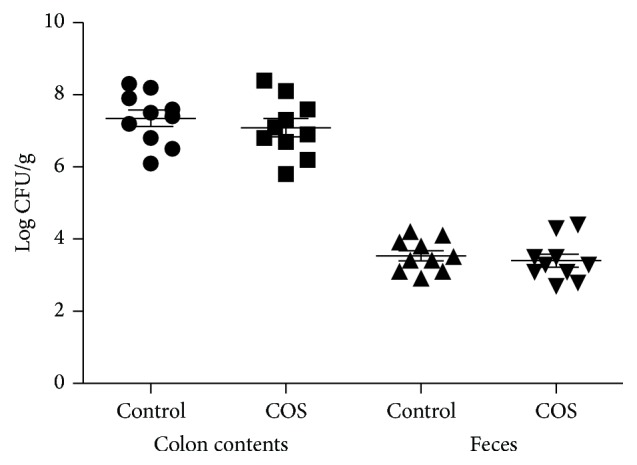
D7 postinfection* C. rodentium* levels in colon contents and feces of infected C57BL/6 mice. Feces and colon contents were gathered before undergoing homogenisation and being plated in serial dilution on LB agar (*n* = 10).

**Figure 2:**
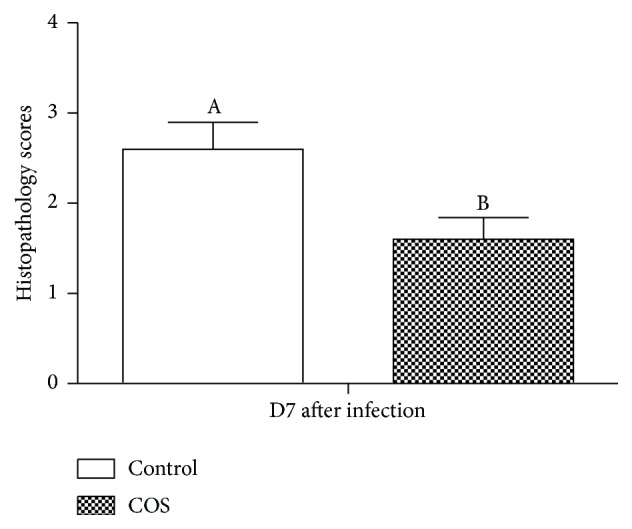
D7 postinfection histopathology scores of each mouse (*n* = 10).

According to the weight measurements, there was no postinfection change in the weight of each mouse in either of the two groups. Additionally, as shown in Figure 1, at D7 after infection, no change was noted in the quantity of* C. rodentium* in the feces or colon contents of each group. However, as shown in Figure 2, a significant increase in colitis severity was observed at D7 after infection amongst the control group of mice compared to the mice provided with COS.

As shown in Table 1, 16S rDNA sequencing was used to analyze the intestinal microbiota once the experiment was completed. The results of 16S rDNA sequencing were used to investigate the effects of COS supplements on histopathology scores. It is clear that the COS-fed mice showed reduced microbiota diversity (as per their feces samples), according to the Shannon and Simpson indices, compared to the control group of mice. The indices also showed that the COS-fed mice showed higher microbiota diversity than the control group of mice based on the colon analysis. Interestingly, it was indicated by the Ace and Chao richness indices that both groups of mice showed similar microbiota community richness in their faecal and colonic samples (Table 1).

**Figure 3:**
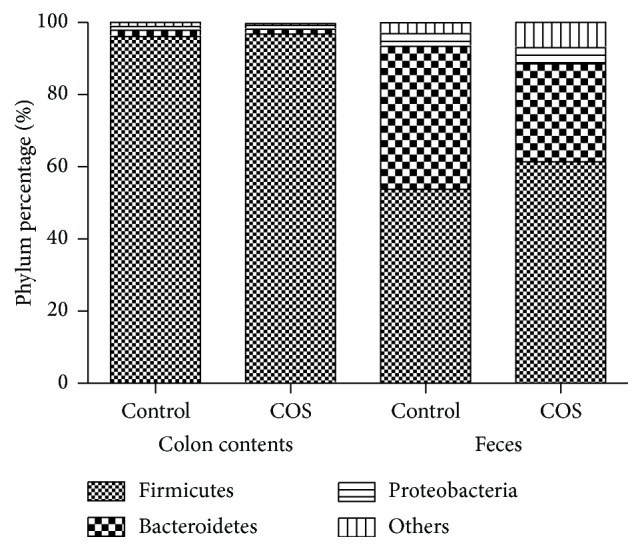
D7 postinfection composition of the intestinal microbiota. Microbial composition in the colon and feces of both groups (*n* = 6). Control mice were given standard drinking water and a basal rodent diet, whilst COS mice were given the same water and diet with the addition of COS at 300 mg/kg.

The RDP classifier was used to perform a taxon-dependent analysis in order to identify the intestinal microbiota's taxonomy. For both groups of mice, the faecal microbiota showed nine phyla, including one candidate division (TM7), whilst the colon microbiota showed seven phyla, including one candidate division (TM7). Additionally, six phyla were found in each of the mice groups. As illustrated in Figure 3, in colon contents, Firmicutes (96.8%) and Proteobacteria (1.3%) were the phyla with the highest percentages in the colon microbiota of the COS-fed mice, whilst in feces Firmicutes (96.1%) and Proteobacteria (1.8%) were the phyla with the highest percentages in the control mice. The percentages of Bacteroidetes (61.4%), Firmicutes (27.1%), and Proteobacteria (4.5%) were the three most abundant in the faecal microbiota of the COS-fed mice, whilst the percentages of Bacteroidetes (53.8%), Firmicutes (39.6%), and Proteobacteria (3.5%) were the most abundant in the control mice.

**Figure 4:**
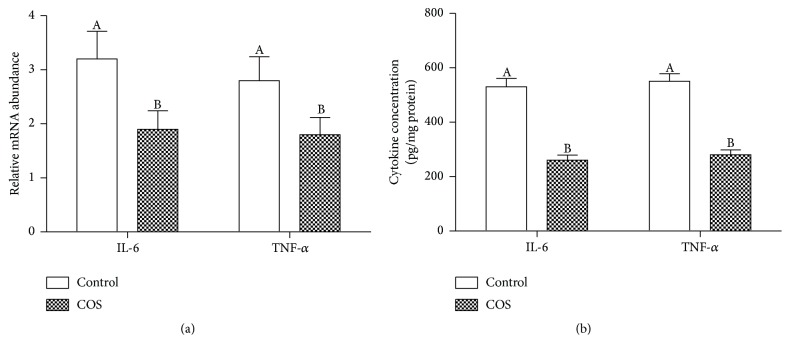
Control and COS group (*n* = 6) mucosal inflammatory responses. (a) RT-PRC evaluation of* IL-6* and* TNF-α* mRNA level. (b) ELISA evaluation of IL-6 and TNF-*α* protein level.

As shown in Figure 4(a), colon analysis of control mice showed significantly higher* IL-6* and* TNF-α* mRNA than the COS-fed mice at D7 after infection. This finding is supported through the ELISA results (Figure 4(b)).

**Figure 5:**
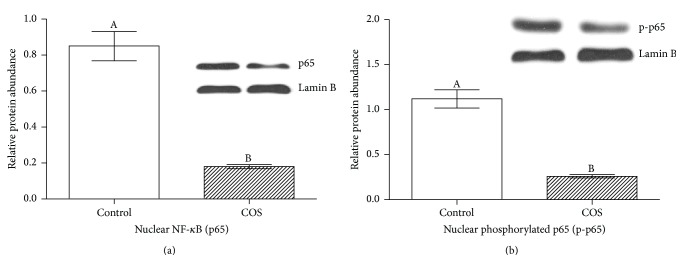
Immunoblotting of (a) nuclear NF-*κ*B (p65) and (b) phosphorylated nuclear NF-*κ*B (p65) in control and COS group (*n* = 6).

Nuclear NF-*κ*B (p65) measurements were taken to identify the activation of NF-*κ*B in the colons of the mice. The purpose of this was to identify whether the beneficial outcomes of COS (i.e., in reducing infection) are supported by the suppression of NF-*κ*B activation pathways. The results showed that the control group of mice had significantly higher nuclear NF-*κ*B (p65) in their colonic samples than the COS-fed group of mice, which suggests that COS suppresses NF-*κ*B activation (see Figure 5).

## 4. Discussion 

*C. rodentium*, which is similar to the human enteropathogenic* E. coli *infection, is an extracellular enteric pathogen that infects mice. It has also been noted that inflammatory bowel disease (IBD) is often modelled using* C. rodentium* in mice. IBD results in intestinal inflammation. Thus, intestinal pathology is regulated by inflammatory regulators such as inducible TNF*α* and IL-6 [6]. Intestinal bacterial communities influence mice's susceptibility and ability to overcome the* C. rodentium* infection, as does the immune response of the mice [23].* E. coli *is commonly modelled by the infection of mice with* C. rodentium* since mice are not affected by EPEC or EHEC.* C. rodentium* has been found in all mice strains but it is rare in human disease. Depending on the strain, an infected mouse can suffer subclinical disease or death after infection [9]. For instance, strains believed to be resistant to developing colitis as a result of* C. rodentium* infection include CD-1 and C57BL/6 mice. On the other hand, it is believed that susceptibility is present amongst mice strains C3H/HeJ and FVB/N [9]. Based on this information, human* E. coli *infection was modelled in this study by infecting C57BL/6 mice with* C. rodentium*.

The results showed that, after infecting the mice with* C. rodentium*, inflammation was resolved more quickly amongst mice receiving 300 mg/kg COS than the control mice. The results noted no impact on the quantity of* C. rodentium* taken from faecal samples, which suggests that the regulating impact of COS is not due to swift pathogenic eradication but rather due to an improvement in microflora diversity. Various biological functions are said to be influenced by such microbiota [19, 20], and it has been associated with cancer [24, 25], cirrhosis of the liver [26], and other diseases. Since Firmicutes has the ability to offer extra energy to the host by fermenting plant polysaccharide to SCFA, this is the reason for obesity being linked to higher concentrations of Firmicutes: Bacteroidetes [27]. At present, the reason behind COS's minimising impact on Firmicutes quantities is unclear. Some studies have also shown that a reduction in intestinal Firmicutes can be achieved through acidic oligosaccharides, a galactooligosaccharides/long-chain fructan solution (GOS/lcF, 9/1), fructooligosaccharides, and other oligosaccharides [28]. It may be that the N-acetyl glucosamine of COS may function as a binding agent that allows COS to influence the attachment of bacteria to the intestine. Given this, research on pigs and chickens indicates that intestinal microbial communities can be impacted by the introduction of COS supplements [18]. Furthermore, intestinal microbiota may change as a result of COS's role as a fermentable substrate for certain bacteria, as this could lower the pH level of the gut and activate natural acid production [26].

Various studies have indicated* in vitro* and* in vivo* anti-inflammatory effects of COS. In the current study, it has been found that IL-6 and TNF-*α* expression in the colon are reduced by COS, whilst microbial flora is impacted by the inflammation [23]. Thus, microbiome changes may be influenced by increased inflammation. Since the faecal samples showed no relationship between* C. rodentium* quantities and the introduction of COS, this finding is contradictory. Consequently, the speed at which* C. rodentium* resolution occurs is related to microflora changes and not inflammation [29]. The results of this study indicate that the effects of COS on the intestines only occur in specific areas of the gut and only take on a specific form. Mice fed with COS were found to have a lower expression of inflammatory cytokines, which is linked to bacterial flora shifts that lead to faster infection recovery.

COS is a d-glucosamine oligomer that cannot be eroded by enzymes in the gut. Thus, COS directly protects the host's intestinal epithelial cells (IEC). Research indicates that IEC expresses various chemokine and cytokine receptors such as TLR4 and other Toll-like receptors [30]. In the IEC, NF-*κ*B-regulated proinflammatory cytokine production is promoted by the interaction between TNF-*α* and their respective receptors, TLR4, TNFR1, and TNFR2 [31]. Studies also show that dysfunction of the intestinal epithelial barrier can occur as a result of pronounced IEC-regulated mucosal inflammatory responses, which are associated with greater TLR4 and TNFR expression [32]. In the current study, it was revealed that a significant reduction in the activation of NF-*κ*B along with colon production of TNF-*α* and IL-6 was achieved through the administration of COS through the basal diet. Since recent studies have found that mice can develop disease sharing similar characteristics to Crohn's as a result of IEC-derived TNF-*α* [32], this indicates that the anti-inflammatory impact of COS could be the result of COS' suppressive impact on the activation of NF-*κ*B and production of proinflammatory cytokines in the IEC.

Overall, it is clear that* in vivo C. rodentium* infection can be attenuated by the introduction of COS into mice's diet. The findings suggest that this is due to the prevention of IL-6 and TNF-*α* expression and NF-*κ*B activation as well as a shift in intestinal microflora. Innovative and successful methods for protecting hosts from* C. rodentium* infection could therefore be achieved through further investigation of the organic carbohydrate oligomer COS.

## Figures and Tables

**Table 1:** Comparative results of 16S rRNA gene libraries' phylotype coverage and diversity at 97% similarity based on pyrosequencing analysis (*n* = 6).

	Number of reads	Number of OUT	Coverage	Richness estimator		Diversity index	
				Ace (95% Cl)	Chao (95% Cl)	Shannon (95% Cl)	Simpson (95% Cl)
Colon							
Control	12,568	37	99.88%	53 (43–86)	58 (43–112)	0.72 (0.69–0.74)	0.68 (0.66–0.70)
COS	12,794	46	99.88%	62 (52–87)	56 (50–81)	0.88 (0.85–0.90)	0.59 (0.58–0.60)
Feces							
Control	11,521	314	99.90%	324 (312–344)	329 (314–357)	4.17 (4.23–4.39)	0.025 (0.024–0.026)
COS	10,956	271	99.43%	304 (289–333)	302 (281–337)	4.05 (4.01–4.08)	0.033 (0.032–0.034)
